# Price-Based Resource Allocation in Wireless Power Transfer-Enabled Massive MIMO Networks

**DOI:** 10.3390/s19153298

**Published:** 2019-07-26

**Authors:** Zhengqiang Wang, Kunhao Huang, Xiaona Yang, Xiaoyu Wan, Zifu Fan, Yongjun Xu

**Affiliations:** 1 School of Communication and Information Engineering, Chongqing University of Posts and Telecommunications, Chongqing 400065, China; 2 Institute of Next Generation Network, Chongqing University of Posts and Telecommunications, Chongqing 400065, China

**Keywords:** massive MIMO, wireless power transfer, Stackelberg game, resource allocation, price

## Abstract

This paper considers the price-based resource allocation problem for wireless power transfer (WPT)-enabled massive multiple-input multiple-output (MIMO) networks. The power beacon (PB) can transmit energy to the sensor nodes (SNs) by pricing their harvested energy. Then, the SNs transmit their data to the base station (BS) with large scale antennas by the harvesting energy. The interaction between PB and SNs is modeled as a Stackelberg game. The revenue maximization problem of the PB is transformed into the non-convex optimization problem of the transmit power and the harvesting time of the PB by backward induction. Based on the equivalent convex optimization problem, an optimal resource allocation algorithm is proposed to find the optimal price, energy harvesting time, and power allocation for the PB to maximize its revenue. Finally, simulation results show the effectiveness of the proposed algorithm.

## 1. Introduction

Due to the increasing demand for data traffic, massive multiple-input multiple-output (MIMO) technology has attracted widespread attention because it can improve spectrum efficiency (SE) and energy efficiency (EE) in mobile communications. Massive MIMO can concentrate the beam in a small area to improve EE and reduce the power consumption of the base station (BS) [1,2]. At the same time, wireless power transfer (WPT) technology has been attracting great attention because it can be used to prolong the life of wireless devices [3,4,5]. Recently, massive MIMO has been considered for WPT systems to improve the transmission distance and efficiency because it can align the radio frequency (RF) signal with the power receivers by exploiting extremely narrow beams [6,7].

Resource allocation for WPT-enabled massive MIMO networks has been studied in [8,9,10,11,12,13]. The performance of WPT in mmWave massive MIMO networks was studied in [8] under rainy or clear conditions. In [9], the overall power transfer efficiency (PTE) and the EE were optimized for a WPT-enabled massive MIMO, where a BS transmitted power to multiple single antenna energy, harvesting users with a massive antenna array. In [10], Fang et al. investigated an energy-harvesting cellular two-way relay network with massive MIMO, and proposed a signal space alignment (SSA)-based simultaneous wireless information and power transfer (SWIPT) protocol. In [11], Lee et al. investigated the low-complexity WPT scheme based on the retrodirective beamforming technique in a multi-user massive MIMO WPT system. In [12], the optimal downlink transmission was studied for massive MIMO-enabled SWIPT systems over Rician fading channels. In [13], an energy efficient resource allocation algorithm was proposed to maximize the EE of the wireless power transfer-enabled massive MIMO sensor networks [14,15,16,17,18] under hardware impairments. In [19], we considered to optimize the EE of wireless powered massive MIMO sensor network based on fractional programming. However, in [8,9,10,11,12,13,19], authors have not considered the resource allocation from the perspective of economics [20,21]. Considering the actual wireless communication scenario, the power beacon (PB) and the energy receiver can belong to different service operators. Therefore, the sensors need to pay for the PB for the charging power. The PB can make a profile by pricing the energy sends to each sensor node. The iteration between the PB and energy receivers can be modeled by Stackelberg game [22,23,24,25]. In [22], Liu et al. studied the pricing problem for operating the antennas in the massive-MIMO enabled wireless virtualized networks by Stackelberg game. However, the wireless power transfer problem is not considered. The utility function for the leader is to the pricing of the antennas to achieve profit maximization. The energy pricing issue is not addressed in [22]. In [23], Sarma et al. studied the Stackelberg game between a BS and a multi-antenna PB for wireless energy harvesting in a multiple sensor nodes (SNs) scenario. An analytical solution is given for a single SN’s case. However, the system model for the PB and the BS is not a massive MIMO system. In [24], Chu et al. investigated a wireless powered communication networks (WPCN)-assisted multi-antenna secure multicasting system, in which a multicast service provider guaranteed secure communication by utilizing the harvested energy from the PB. In [25], the price-based resource allocation algorithm was investigated for energy harvesting massive MIMO system by a Stackelberg game. However, the PB was equipped with a single antenna in the system model [25]. Moreover, the algorithm proposed in [25] cannot be used for the PB with massive MIMO.

### 1.1. Summary of Contribution

We investigate the price-based resource allocation for WPT-enabled massive MIMO sensor networks. In the proposed system model, the sensors are powered by the PB with a large scale of antennas by pricing and then transmiting data to the BS with massive MIMO. The main contributions of this paper are summarized as follows:

- We model the interaction between the PB and SN in WPT-enabled massive MIMO system as a Stackelberg game. The revenue problem of the PB is transformed into a non-concave function of the transmit power and the harvesting time of PB by backward induction.
- We prove that the optimal total transit power for PB should equal the maximum power. The revenue of the PB is converted into an equivalent convex resource allocation problem by the optimal condition of the PB’s total transmit power.
- We give the optimal prices for the PB and the closed-form power allocation for the SNs. The complexity of the proposed algorithm is analyzed. Simulation results are presented to verify the effectiveness of the proposed algorithm.

### 1.2. Organization

The rest of this paper is organized as follows: In Section 2, the system model is given and the Stackelberg game problem is formulated. In Section 3, the optimal price-based resource allocation algorithm is proposed based on convex optimization. Simulation results are given in Section 4 to show the impact of the maximum power on the revenue of the PB and SNs. Finally, conclusions are drawn in Section 5. Proofs and abbreviations are presented to the Appendices.

## 2. System Model and Problem Formulation

### 2.1. System Model

**Figure 1:**
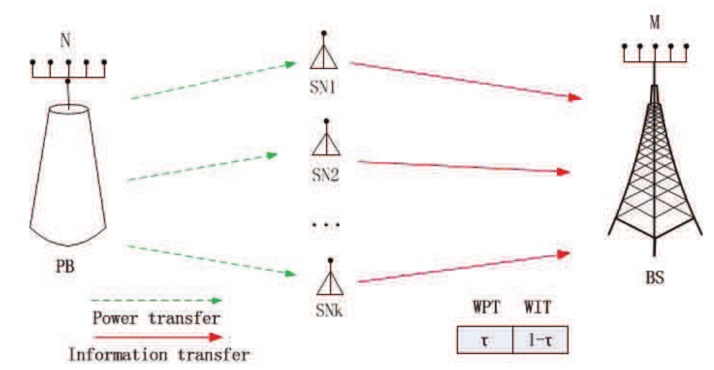
System Model.

As shown in Figure 1, the system consists of a BS with *M* antennas, a PB with *N* antennas and *K* single antenna SNs, where min{M,N}≫K is held. The noise vector at the BS is $n∼CN(0_{M},\sigma^{2}I_{M}).$ It is assumed that the BS knows the perfect channel state information (CSI) and uses a zero-forcing (ZF) receiver. The channel state information can be obtained by channel estimation with pilot signals from the SNs to the BS [26,27]. The ZF receiver is used because M≫K is held. Moreover, ZF is low complexity receivers, which has a better ability to cancel multi-user interference compared to the maximum ratio combining (MRC) receiver [28]. Harvest-then-transmit protocol is used for the proposed system [29]. For the sake of simplicity, the time slot is normalized to be 1 and divided into two parts. In the first part of the time, the PB transmits energy to the SN. Then, the SN sends information to BS in the second part of the time.

The strategy between the PB and the SNs is modeled as a Stackelberg game. PB is the leader in this game, and SN is the follower in the game. PB charges the *k*-th sensor $\lambda_{K}$ per unit of power to maximize its own revenue. After the PB broadcasts the price for each SN, the SN will choose a suitable power to maximize its own utility. In the first time τ for the wireless energy transmission phase, the energy harvested by the *k*-th sensor is given by [30].

(1) $$E_{k}\left(p_{k}\right)=\tau \beta_{k}\left(p_{k}N+\sum_{j\neq k}p_{j}\right)$$

where $\beta_{k}$ represents the large-scale fading from the PB to the *k*-th sensor node, and $p_{k}$ is the energy allocation for SN *k*. In the second time 1−τ for the data transmission phase, the average power of senor *k*-th SN can be expressed as $\frac{\xi_{k}E_{k}\left(p_{k}\right)}{\left(1-\tau \right)}$ because it uses all the harvesting power in the first time τ with harvest-then-transmit protocol [29], where we have assumed that the circuit power consumption of the SN can be negligible compared to its uplink transmit power as [31,32,33,34]. Then, the achievable throughput of the *k*-th SN under ZF is given by [35]:

(2) $$\begin{array}{cc} D_{k}\left(p_{k}\right) & =\left(1-\tau \right)\log\left(1+\frac{\left(M-K\right)\alpha_{k}\xi_{k}E_{k}\left(p_{k}\right)}{\sigma^{2}\left(1-\tau \right)}\right)\\ & =\left(1-\tau \right)\log\left(1+\frac{\tau \left(M-K\right)\alpha_{k}\beta_{k}\xi_{k}\left(p_{k}N+\sum_{j\neq k}p_{j}\right)}{\left(1-\tau \right)\sigma^{2}}\right) \end{array}$$

where $\alpha_{k}$ is the large-scale fading factor of the *k*-th sensor to the BS, $\xi_{k}$ is the energy conversion efficiency of the *k*-th SN. $\sigma^{2}$ is the background noise at the BS.

### 2.2. Problem Formulation

The problem of the PB is as follows:

(3) $$\begin{array}{c} \max U\left(\lambda ,p,\tau \right)=\tau \sum_{k=1}^{K}\lambda_{k}p_{k}\\ s.t.\;\;\;\lambda_{k}\geq 0,k=1,\cdots ,K,\\ \;\;\;\;\;\;\sum_{k=1}^{K}p_{k}\leq P_{\max},\\ \;\;\;\;\;\;\;0\leq \tau \leq 1. \end{array}$$

The optimization variables for Equation (3) are λ and τ. $P_{\max}$ is the maximum transmit power of PB, λ is the price vector for all SNs such that $\lambda ={\left[\lambda_{1},\lambda_{2},\cdots \lambda_{K}\right]}^{T}$, where $\lambda_{k}$ is the price of harvesting unit power from the PB by the *k*-th sensor, $p={\left[p_{1},p_{2},\cdots ,p_{k}\right]}^{T}$ is power vector that SNs purchased from the PB for the given price λ. We have used the non-uniform pricing model [36] for the PB charging each SN, which is different from the quadratic model used in [24].

The utility of the *k*-th sensor contains two parts: one is the income due to data transmission to the BS and the other one is the payment to the PB for the energy harvesting. Therefore, the revenue of the *k*-th SN is given by:

(4) $$\begin{array}{c} \max U_{k}\left(\lambda ,p,\tau \right)=\left(1-\tau \right)\log\left(1+\frac{\tau \left(M-K\right)\alpha_{k}\beta_{k}\xi_{k}\left(p_{k}N+\sum_{j\neq k}p_{j}\right)}{\left(1-\tau \right)\sigma^{2}}\right)-\lambda_{k}p_{k}\tau \\ s.t.\;\;p_{k}\geq 0 \end{array}$$

The optimization variable for Equation (4) is $p_{k}$. The first item in the objective function of Equation (4) is the rate of *k*-th SN at the BS, and the second item is the payment to the PB. We have defined the income for *K* SNs to maximizing the rate of each user, which is different from the utility function in [24]. In [24], only one subproblem is considered by maximizing multicast secrecy rate of the system.

## 3. Optimal Price-Based Resource Allocation Algorithm

This section gives the optimal-price based resource allocation algorithm for the system model by a backward induction method. First, we present the relationship between price $\lambda_{k}$ and transmit power $p_{k}$ for a given energy harvesting time. Then, the PB’s revenue is expressed as a function of the transmit power and energy harvesting time. The objective function is proved to be equivalent to a convex optimization for a given energy harvesting time. Then, the transmit power and energy harvesting time can be obtained by convex optimization and alternating optimization.

The relationship between PB’s price $\lambda_{k}$ and the SN’s harvesting power $p_{k}$ is given by the following lemma.

**Lemma** **1.** *For a given τ∈(0,1), let $p_{1},\cdots ,p_{k}$ be the optimal buying power allocation of the SNs when the PB charges the k-th SN price $\lambda_{k}$ such that $\frac{\left(1-\tau \right)\left(M-K\right)N\alpha_{k}\beta_{k}\xi_{k}}{\left(1-\tau \right)\sigma^{2}+\tau \left(M-K\right)\alpha_{k}\beta_{k}\xi_{k}P_{\max}N}\leq \lambda_{k}\leq \frac{\left(M-K\right)N\alpha_{k}\beta_{k}\xi_{k}}{\sigma^{2}}$ is held, then the relationship between price and the transmit power satisfy the following equations:*

(5) $$\lambda_{k}=\frac{\left(1-\tau \right)\left(M-K\right)N\alpha_{k}\beta_{k}\xi_{k}}{\left(1-\tau \right)\sigma^{2}+\tau \left(M-K\right)\alpha_{k}\beta_{k}\xi_{k}\left(p_{k}N+\sum_{j\neq k}p_{j}\right)}$$

**Proof** **(Proof** **of** **Lemma** **1).** See Appendix A □.

From (A1), we know that the buying power of the *k*-th SN will be zero when $\lambda_{k}>\frac{\left(M-K\right)N\alpha_{k}\beta_{k}\xi_{k}}{\sigma^{2}}$ is held. Moreover, the buying power of the *k*-th SN will be larger than $P_{max}$ if $\lambda_{k}<\frac{\left(1-\tau \right)\left(M-K\right)N\alpha_{k}\beta_{k}\xi_{k}}{\left(1-\tau \right)\sigma^{2}+\tau \left(M-K\right)\alpha_{k}\beta_{k}P_{\max}N}$ is held. Therefore, we only need to consider the price for user *k* such that $\frac{\left(1-\tau \right)\left(M-K\right)N\alpha_{k}\beta_{k}\xi_{k}}{\left(1-\tau \right)\sigma^{2}+\tau \left(M-K\right)\alpha_{k}\beta_{k}\xi_{k}P_{\max}N}\leq \lambda_{k}\leq \frac{\left(M-K\right)N\alpha_{k}\beta_{k}\xi_{k}}{\sigma^{2}}$ is held.

Using Lemma 1, substitute Equation (5) into Equation (3), the revenue maximization problem of the PB can be rewritten as follows.

(6) $$\begin{array}{c} \max_{p,\tau }f\left(p,\tau \right)=\sum_{k=1}^{K}\frac{\left(1-\tau \right)\tau \left(M-K\right)N\alpha_{k}\beta_{k}\xi_{k}p_{k}}{\left(1-\tau \right)\sigma^{2}+\tau \left(M-K\right)\alpha_{k}\beta_{k}\xi_{k}\left(p_{k}N+\sum_{j\neq k}p_{j}\right)}\\ s.t.\;\;p_{k}\geq 0,k=1,\ldots ,K,\\ \;\;\;\;\;\sum_{k=1}^{K}p_{k}\leq P_{\max},\\ \;\;\;\;0\leq \tau \leq 1. \end{array}$$

Because the objective function in Equation (6) is non-concave with respect to τ and *p*, Equation (6) is a non-convex optimization problem. It is difficult to give the optimal time allocation and power allocation by Equation (6). First, we give the optimal power allocation for a given time allocation. Then, we prove that Equation (6) is a convex optimization problem with respect to τ for a given power allocation. Therefore, the bisection method can be used to find the optimal time allocation for the given power allocation. Finally, we give an iteration price-based resource allocation algorithm to maximize the revenue of the PB. For a given τ and let $A_{k}=\frac{\left(M-K\right)\alpha_{k}\beta_{k}\xi_{k}}{\sigma^{2}}$, Equation (6) can be rewritten as follows.

(7) $$\begin{array}{c} \max_{p}\sum_{k=1}^{K}\frac{A_{k}N\tau p_{k}}{1+\frac{\tau }{1-\tau }A_{k}\left(p_{k}N+\sum_{j\neq k}^{}p_{j}\right)}\\ s.t\;\;\sum_{k=1}^{K}p_{k}\leq P_{\max},\\ \;\;\;\;\;p_{k}\geq 0,\;k=1,\ldots k. \end{array}$$

where $A_{k}=\frac{\left(M-K\right)\alpha_{k}\beta_{k}\xi_{k}}{\sigma^{2}}$.

**Lemma** **2.** *Let $\left(p_{1},\cdots ,p_{K}\right)$ be the optimal solution to Equation *(7)* for a given $P_{max}$ and τ, then the following condition is held:*

(8) $$\sum_{k=1}^{K}p_{k}=P_{\max}$$

**Proof** **(Proof** **of** **Lemma** **2).** See Appendix A. □

Using Lemma 2, Equation (7) is equivalent to the following problem:

(9) $$\begin{array}{c} \max_{P}\sum_{k=1}^{K}\frac{A_{k}N\tau p_{k}}{1+\frac{\tau }{1-\tau }A_{k}\left(p_{k}\left(N-1\right)+P_{\max}\right)}\\ s.t\;\;\sum_{k=1}^{K}p_{k}=P_{\max},\\ \;\;\;\;\;p_{k}\geq 0,\;k=1,\ldots k. \end{array}$$

It is easy to prove that Equation (7) is a concave function for a fixed time τ. Using the Lagrangian multiplier method to solve Equation (7) for a fixed τ, we give the optimal power allocation as follows.

**Theorem** **1.** *Let $p_{1},\cdots ,p_{K}$ be the optimal solution to Equation *(9)* for a fixed τ, then $p_{k}\left(k=1,\cdots ,K\right)$ is given as follows:*

(10) $$p_{k}=\frac{1}{N-1}{\left(\left(\sqrt{\frac{A_{k}N\tau \left(1+\frac{\tau }{1-\tau }A_{k}P_{\max}\right)}{\xi }}-1\right)\frac{1-\tau }{A_{k}\tau }-P_{\max}\right)}^{+}$$

*where ${\left(x\right)}^{+}$ is defined as max(0,x), ξ is the solution to the following equation:*

(11) $$\sum_{k=1}^{K}\frac{{\left(\left(\sqrt{\frac{A_{k}N\tau \left(1+\frac{\tau }{1-\tau }A_{k}P_{\max}\right)}{\xi }}-1\right)\frac{1-\tau }{A_{k}\tau }-P_{\max}\right)}^{+}}{N-1}=P_{\max}$$

**Proof** **(Proof** **of** **Theorem** **1).** See Appendix A. □

The solution ξ to Equation (11) can be obtained by the bisection method. Moreover, using the same method as [37], we can obtain the closed-form solution for ξ as follows.

**Theorem** **2.** *Assuming that all the SNs are sorted such that $A_{1}\leq A_{2}\cdots \leq A_{K}$, the solution ξ to Equation *(11)* is given by*

(12) $$\xi =\frac{{\left(\sum_{k=i}^{K}\sqrt{A_{k}N\tau \left(1+\frac{\tau }{1-\tau }A_{k}P_{\max}\right)}\frac{1-\tau }{A_{k}\tau }\right)}^{2}}{{\left(\left(K-i+N\right)P_{\max}+\sum_{k=i}^{K}\frac{1-\tau }{A_{k}\tau }\right)}^{2}}$$

*where $i=\min_{1\leq j\leq K}\left\{j|a_{j}\left(\tau \right)<b_{j}\left(\tau \right)\right\}$, $a_{j}\left(\tau \right)$ and $b_{j}\left(\tau \right)$ is defined as $a_{j}\left(\tau \right)=\frac{{\left(\sum_{k=j}^{K}\sqrt{A_{k}N\tau \left(1+\frac{\tau }{1-\tau }A_{k}P_{\max}\right)}\frac{1-\tau }{A_{k}\tau }\right)}^{2}}{{\left(\left(K-j+N\right)P_{\max}+\sum_{k=j}^{K}\frac{1-\tau }{A_{k}\tau }\right)}^{2}}$ and $b_{j}\left(\tau \right)=\frac{A_{j}N\tau }{\left(\frac{P_{\max}A_{j}\tau }{1-\tau }+1\right)}.$*

**Proof** **(Proof** **of** **Theorem** **2).** See Appendix A. □

From Theorems 1 and 2, we can get the optimal power allocation for a given τ. For a given power allocation, using Lemma 2, the optimization problem Equation (7) can be rewritten as the following time allocation problem.

(13) $$\begin{array}{c} \max_{\tau }g\left(\tau \right)=\sum_{k=1}^{K}\frac{A_{k}N\tau p_{k}}{1+\frac{\tau }{1-\tau }A_{k}\left(p_{k}\left(N-1\right)+P_{\max}\right)}\\ s.t.\;\;0\leq \tau \leq 1. \end{array}$$

Because $\frac{d^{2}g\left(\tau \right)}{d\tau^{2}}=\sum_{k=1}^{K}-\frac{2\;N\;{A_{k}}^{2}\;\;p_{k}\;\left(P_{\max}+\left(N-1\right)\;\;p_{k}\right)}{{\left(A_{k}\;\;\tau \left(P_{\max}+p_{k}\;\left(N-1\right)\right)+1-\tau \right)}^{2}}\leq 0$, and the constraint of Equation (13) is linear constraint, Equation (13) is a convex optimization problem. The first order of $g\left(\tau \right)$ is given by $\frac{dg\left(\tau \right)}{d\tau }=-\sum_{k=1}^{K}\frac{N\;A_{k}\;p_{k}\;\left(2\;\tau -\tau^{2}-A_{k}\;\;\;p_{k}\;\tau^{2}+P_{\max}\;A_{k}\;\;\tau^{2}+N\;A_{k}\;\;p_{k}\;\tau^{2}-1\right)}{{\left(\;A_{k}\;\;\tau \left(P_{\max}+p_{k}\;\left(N-1\right)\right)+1-\tau \right)}^{2}}$. Moreover, we have $\frac{dg\left(\tau \right)}{d\tau }{|}_{\tau =0}=N\sum_{k=1}^{K}A_{k}\;\;p_{k}>0$ and $\frac{dg\left(\tau \right)}{d\tau }{|}_{\tau =1}=-\sum_{k=1}^{K}\frac{N\;A_{k}\;p_{k}\;}{\left(P_{\max}\;A_{k}\;+\;\left(N-1\right)A_{k}\;\;p_{k}\;\right)}<0$. Using the first order optimal condition, the optimal solution to Equation (13) can be obtained given by the following theorem.

**Theorem** **3.** *For a fixed power allocation $p_{1},\cdots ,p_{K},$ the optimal time τ to Equation *(13)* is the unique solution to the following equation:*

(14) $$\sum_{k=1}^{K}\frac{N\;A_{k}\;p_{k}\;\left(2\;\tau -\tau^{2}-A_{k}\;\;\;p_{k}\;\tau^{2}+P_{\max}\;A_{k}\;\;\tau^{2}+N\;A_{k}\;\;p_{k}\;\tau^{2}-1\right)}{{\left(\;A_{k}\;\;\tau \left(P_{\max}+p_{k}\;\left(N-1\right)\right)+1-\tau \right)}^{2}}=0$$

For a given power allocation $p_{1},\cdots ,p_{K}$, the optimal time τ to the Equation (14) can be found by the bisection method in [0,1]. Based on Theorems 1–3, we can solve the Equation (7) by power allocation and time allocation iteratively. By Lemma 1, and Theorems 1–3, we give an optimal price-based resource allocation algorithm as Algorithm 1.

The optimal problem is solved by time and power allocation separately using block Gauss-Seidel method. The total variables can be viewed as two blocks. One block is time, and another is power. Since the sets for time and power are decouple and convex, and subproblems for time optimization and power optimization have a unique optimal solution, the proposed algorithm is globally convergent [38]. Moreover, we will show in the simulation parts that the proposed algorithm is convergent to optimal solution by exhaustive search method, which searches the optimal energy harvesting time in [0,1] exhaustively to find the optimal solution of problem Equation (7) because we have known the optimal power allocation for a fixed time.

Finally, we do the complexity analysis of the proposed algorithm. From Algorithm 1, the complexity of the proposed algorithm only depends on the complexity of power allocation in Equation (10) and time allocation Equation (14). From Theorem 2, we can see that power allocation for SNs can be expressed by a closed-form with parameter ξ, which can be obtained by at most *K* times compare comparison. So the complexity of the power allocation is O(K), where *K* is the number of SNs. The complexity of the time allocation Equation (14) is obtained by the bisection method, which needs at most $O(log\left(\frac{1}{\varepsilon }\right))$ time iterations, where ε is the tolerance value. Therefore, the computational complexity the proposed algorithm is given by $O(Klog\left(\frac{1}{\varepsilon }\right))$.

**Algorithm 1** Optimal Price-based Resource Allocation Algorithm (OPRAA).**Initialization:**$A_{k}$ such that $A_{1}\leq A_{2}.\cdots \leq A_{K}$, maximum iterative number $s_{\max}$, convergence threshold ε, iterative number s=1, $\tau^{\left(s\right)}=\frac{1}{2}$, $p^{\left(s\right)}=\left(P_{\max}/K,\cdots ,P_{\max}/K\right)$, 
**repeat**

s=s+1
power allocation: For a given time allocation $\tau^{\left(s-1\right)}$, update the power of all user $p^{\left(s\right)}=\left(p_{1}^{\left(s\right)},\cdots ,p_{K}^{\left(s\right)}\right)$, where $p_{k}^{\left(s\right)}$ is power of user *k* in iteration *s*, which is obtained by Equation (10). time allocation: For a given power allocation $p^{\left(s\right)}$, $\tau^{\left(s\right)}$ is the solution to Equation (14) obtained by bisection method,**until**$s=s_{\max}$ or $f\left(p^{\left(s\right)},\tau^{\left(s\right)}\right)-f\left(p^{\left(s-1\right)},\tau^{\left(s-1\right)}\right)<\varepsilon$**output** the energy harvesting time is given by $\tau =\tau^{s}$, the buying energy power for user *k* is $p_{k}=p_{k}^{\left(s\right)}\left(k=1,\cdots ,K\right)$, the price for user *k* is given by $\lambda_{k}=\frac{\left(1-\tau \right)\left(M-K\right)N\alpha_{k}\beta_{k}\xi_{k}}{\left(1-\tau \right)\sigma^{2}+\tau \left(M-K\right)\alpha_{k}\beta_{k}\xi_{k}\left(p_{k}N+\sum_{j\neq k}p_{j}\right)}$.

## 4. Simulation Results

In this section, we give some simulation results to demonstrate the performance of the proposed optimal price-based resource allocation algorithm (OPRAA). To gain the insight of the impact of the energy harvesting time on the system performance, we also show the performance of the equal time resource allocation algorithm (ETRAA) which sets the energy harvesting time and the data transmit time. The power allocation for each SN in ETRAA is also obtained by the proposed algorithm without the energy harvesting time optimization. The number of antennas of the PB is N=100, the number of SNs is K=10, the coordinates of the BS and PB are (30,0) m and (−30,0) m, and the SNs are randomly distributed on the [−25,25]×[−25,25] m. The large-scale fading from PB to the SN and the BS to the SN are respectively $\beta_{k}=\frac{1}{10^{3}}m_{k}^{-3}$, $\alpha_{k}=\frac{1}{10^{3}}l_{k}^{-3}$, where $m_{k}$ is the distance from PB to sensor *k*, and $l_{k}$ is the distance from BS to SN *k*. Background noise is $\sigma^{2}=10^{-10}W$. Simulation results are averaged over $10^{3}$ independent channel realizations.

**Figure 2:**
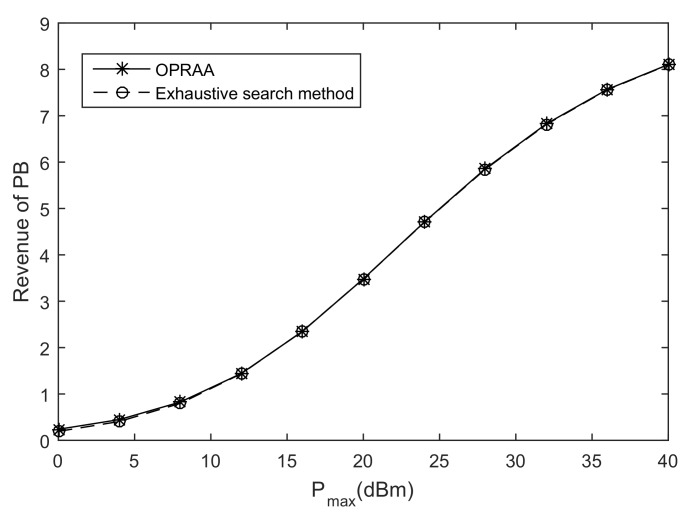
Revenue of PB.

Figure 2 shows the performance of the PB by the proposed OPRAA with the exhaustive search method to find the optimal solution to Equation (6) when M=100, N=100, and K=10. We can see that the OPRAA can convergence to the exhaustive search method. We find the result of the OPRAA converges to exhaustive search method through many times simulations for different *M*, *N* and *K*. Therefore, there are no of the performance loss by the proposed algorithm with respect to optimal solution obtained by exhaustive search method.

**Figure 3:**
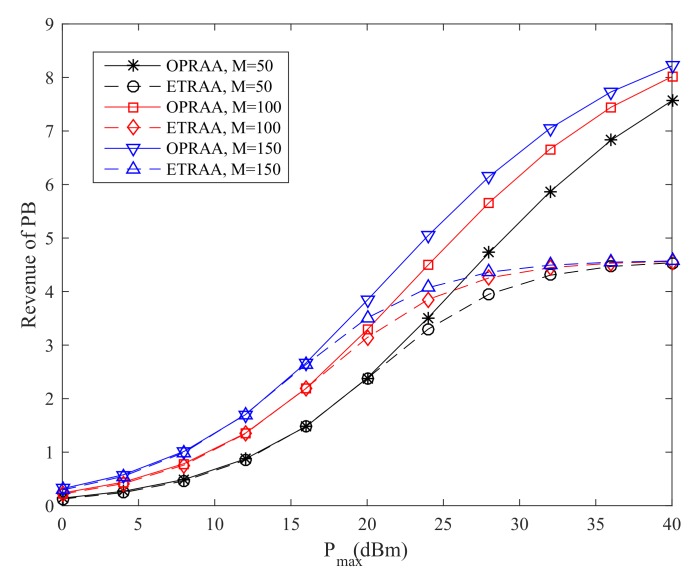
Revenue of PB.

Figure 3 shows the revenue of the PB by the OPRAA and ETRAA with different maximum transmit power at the PB. We can see the revenue of the PB increases with the maximum transmit power for two algorithms. This is because the PB has more power to allocate to each SN as the maximum transmit power increases. Moreover, the revenue obtained by OPRAA is better than ETRAA for the same number of antennas at the BS. As the number of antennas increases at the BS, the performance of both algorithms also increases due to the multi-antenna diversity. When the maximum transmit is 40 dBm and the number of antennas is 150 at the BS, the revenue of the PB with OPRAA increases 79.7% than the ETRAA method. This is because the PB can have more pricing strategies in OPRAA by using time allocation for energy harvesting to obtain more revenue from SNs compared with ETRAA. From Figure 3, the sum revenue of the PB can be improved significantly by harvesting time optimization as the maximum power at the PB increases.

**Figure 4:**
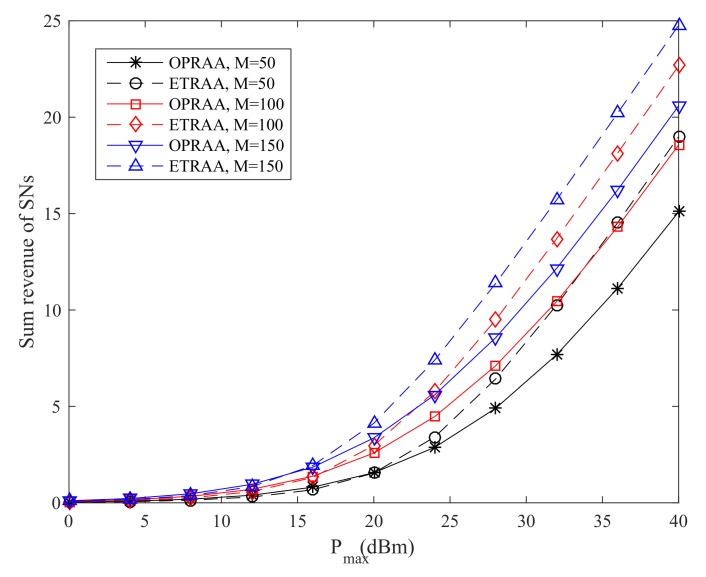
Sum Revenue of SNs.

In Figure 4, the sum revenue of SNs increases as the maximum transmit power increases. This is because the SNs can harvest more power for their data transmission. Moreover, the performance with ETRAA is better than the OPRAA. When $P_{max}$ equals 40 dBm and the number of antennas is 150 at the BS, the sum revenue obtained OPRAA is 16.8% less than the ETRAA method. This is because the OPRAA is optimized from the revenue maximization problem for the PB. The strategies for the PB to maximize its revenue by time optimization for energy harvesting will lead the SNs to pay more expenses to buy energy from the PB.

**Figure 5:**
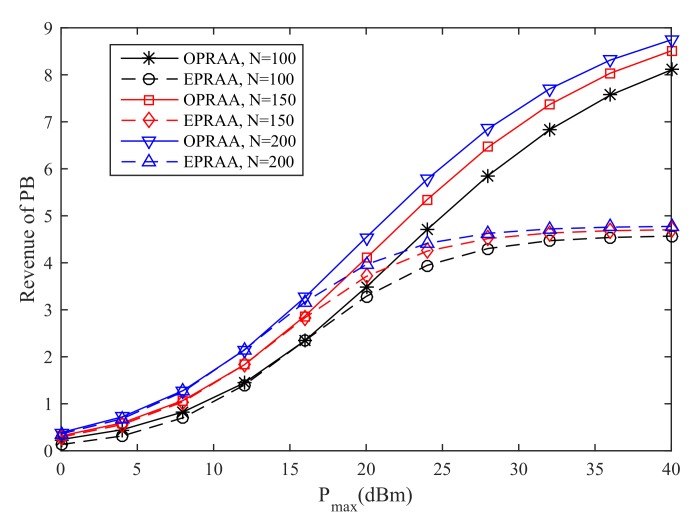
Sum Revenue of SNs.

In Figure 5, the revenue of the PB for the different number of *N* versus the maximum transmit power is given. We can see that the revenue obtained by OPRAA is better than ETRAA for the same number of antennas at the PB under the same $P_{max}$. As the number of antennas increases at the PB, the performance of both algorithms because the PB can have more energy allocation strategies with more antennas.

**Figure 6:**
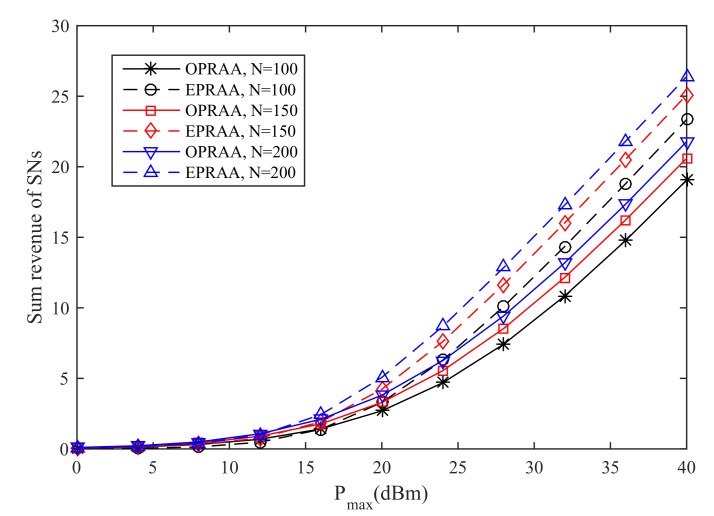
Sum Revenue of SNs.

In Figure 6, the sum revenue of SNs for the different number of *N* is given, where M=100 and K=10. As the number of antennas increases, the sum revenue of SNs obtained by two algorithms increases. When $P_{max}$ equals 40 dBm and the number of antennas is 150 at the PB, the sum revenue achieved by OPRAA is 17.9% less than the ETRAA method.

**Figure 7:**
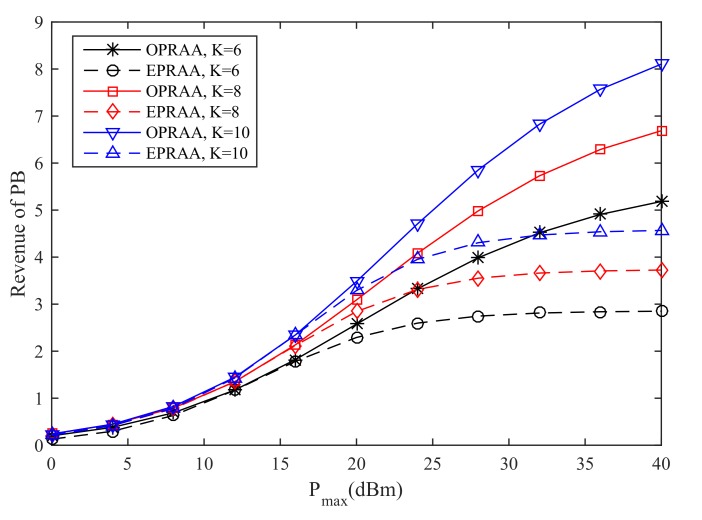
Sum Revenue of SNs.

In Figure 7, the revenue of the PB for the different number of *K* versus the maximum transmit power is given, where M=100 and N=100. We can see that the revenue obtained by OPRAA is better than ETRAA for the same number of users at the PB under the same $P_{max}$. As the number of users increases, the revenue of two algorithms increases due to the multi-user diversity.

**Figure 8:**
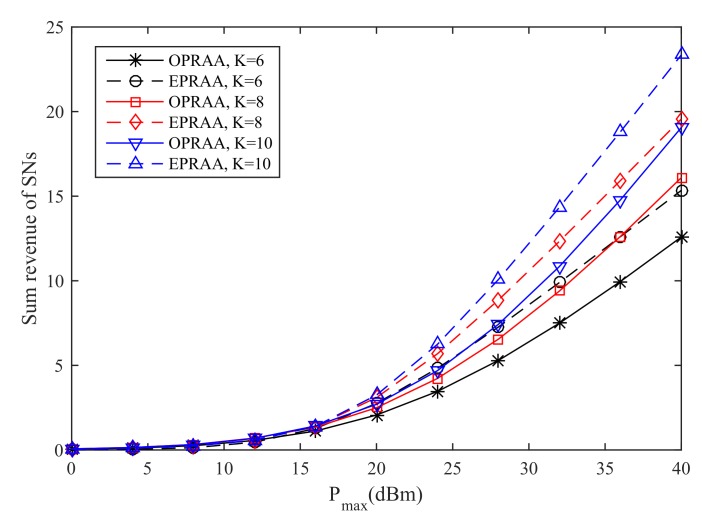
Sum Revenue of SNs.

Figure 8 show the sum revenue of the SNs for the different number of *K* versus the maximum transmit power is given, where M=100 and N=100. We can see that the revenue obtained by ETRAA is better than OPRAA for the same number of users at the PB under the same $P_{max}$, which has the same reason as Figure 3. As the number of users increases, the revenue obtained by both algorithms increases due to the multi-user diversity.

From the above discussion, we can see that the energy harvesting time optimization for the proposed game can improve the revenue of the PB, but this will cause the performance loss of SNs in terms of sum revenue. Therefore, different energy harvesting time in the proposed Stackelberg game can be used to balance the revenue of the PB and the sum revenue of the SNs.

## 5. Conclusions

In this paper, we investigate price-based resource allocation in wireless power transfer-enabled massive MIMO sensor networks by a Stackelberg game. The optimal price, energy harvesting time, and power allocation for the PB to maximize its revenue is given based on an equivalent convex optimization problem. A price-based resource allocation algorithm is proposed to maximize the revenue of the PB, which can converge to be the optimal solution obtained by the exhaustive search method. Simulation results show that the proposed algorithm can achieve better performance as the maximum power at the PB or the number of the antennas at the BS increases. Moreover, the energy harvesting time optimization has the opposite effect on the performance of the PB and SNs.

The sub-problem for each SN is considered as a non-cooperative game to find its optimal strategy for the proposed system model. However, some SNs may cooperate to form a coalition to biding the resource from the PB to have a better outcome in the practical engineering application scenario. We aim that our future work is to model the strategy between the SNs and the PB when some SNs are contingent cooperator. Other approaches such as contingent theory [39] could be useful to model the cooperation among the SNs. We have ignored the influence of the transceiving circuit power consumption for SNs because we have assumed the SNs are the lower-powered device such as sensors deployed for the Internet of Things (IoT). It is also an essential issue for extending our system model under the consideration of the transceiving circuit power consumption as [9,40,41,42,43]. Moreover, the SNs have the same priority in our game for transmitting data to the PB, which may lead to the near-far problem. We can use different priority factor for the SNs to overcome the near-far problem as [37]. If a user is far from the BS, we can give a higher priority for its utility function to motivate it to buy more energy. Last but not least, we have assumed that the BS has knowledge about perfect CSI for all SNs to handle the problem tractability. The pilot training interval is omitted in the system model because pilot training time is much less than the data transmission. However, the CSI obtained by the uplink pilot from the SNs to BS can be imperfect in the actual communication environment by channel estimation error. The revenue obtained by the PB in this paper under the perfect CSI can be viewed as an upper bound for the imperfect CSI case. One of our future work is to consider adding the channel estimation time slot in the system model. We will jointly optimize the energy harvesting time, pilot time, and data transmission time together. The model used in [30] with pilot training for channel estimation before wireless information transfer can be used for the SNs and the BS.

## Abbreviations

The following abbreviations are used in this manuscript:

WPT	wirelesspowertransfer
MIMO	massive multiple-input multiple-output
SNs	sensor nodes
PB	power beacon
BS	base station
EE	energy efficiency
SE	spectrum efficiency
RF	radio frequency
PTE	power transfer efficiency
SSA	signal space alignment
SWIPT	simultaneous wireless information and power transfer
WPCN	wireless powered communication networks
ZF	zero-forcing
MRC	maximum ratio combining
OPRAA	optimal price-based resource allocation algorithm
ETRAA	equal time resource allocation algorithm

## Appendix A

**Proof** **(Proof** **of** **Lemma** **1).** Because $\frac{d^{2}U_{k}}{dp_{k}^{2}}=\frac{-\left(1-\tau \right)\tau^{2}{\left(M-K\right)}^{2}N^{2}{\alpha_{k}}^{2}{\beta_{k}}^{2}{\xi_{k}}^{2}}{{\left(\left(1-\tau \right)\sigma^{2}+\tau \left(M-K\right)\alpha_{k}\beta_{k}\xi_{k}\left(p_{k}N+\sum_{j\neq k}p_{j}\right)\right)}^{2}}<0$, problem Equation (4) is a convex optimization problem. Use the optimal condition for the *k*-th SN in Equation (4), we have

(A1) $$\frac{dU_{k}}{dp_{k}}=\frac{\left(1-\tau \right)\tau \left(M-K\right)N\alpha_{k}\beta_{k}\xi_{k}}{\left(1-\tau \right)\sigma^{2}+\tau \left(M-K\right)\alpha_{k}\beta_{k}\xi_{k}\left(p_{k}N+\sum_{j\neq k}p_{j}\right)}-\lambda_{k}\tau =0$$

That is $\lambda_{k}=\frac{\left(1-\tau \right)\left(M-K\right)N\alpha_{k}\beta_{k}\xi_{k}}{\left(1-\tau \right)\sigma^{2}+\tau \left(M-K\right)\alpha_{k}\beta_{k}\xi_{k}\left(p_{k}N+\sum_{j\neq k}p_{j}\right)}$ is held. Thus, Lemma 1 is proved. □

**Proof** **(Proof** **of** **Lemma** **2).** We prove it by contradiction. Let $\left(p_{1},.\cdots p_{K}\right)$ be the optimal solution to Equation (7) such that $\sum_{k=1}^{K}p_{k}^{}<P_{max}$ is held. Let $a=\frac{P_{\max}}{\sum_{k=1}^{K}p_{k}}>1$ and $p_{k}^{*}=ap_{k},k=1,\cdots ,K$. Then, we have $\sum_{k=1}^{K}p_{k}^{*}=P_{\max}$ is held. Moreover, the following condition is satisfied:

(A2) $$\sum_{k=1}^{K}\frac{A_{k}N\tau p_{k}^{*}}{1+\frac{\tau }{1-\tau }A_{k}\left(p_{k}^{*}N+\sum_{j\neq k}^{}p_{j}^{*}\right)}\;>\;\sum_{k=1}^{K}\frac{A_{k}N\tau p_{k}}{1+\frac{\tau }{1-\tau }A_{k}\left(p_{k}N+\sum_{j\neq k}^{}p_{j}\right)}\;$$

This contradicts the assumption that $\left(p_{1},\cdots ,p_{K}\right)$ is the optimal solution to Equation (7). Thus, Lemma 2 is proved. □

**Proof** **(Proof** **of** **Theorem** **1).** Let $p=(p_{1},\cdots p_{K})$, the Lagrangian function of Equation (9) is given as follows

(A3) $$\begin{array}{c} L\left(p,\xi \right)=\sum_{k=1}^{K}\frac{A_{k}N\tau p_{k}}{1+\frac{\tau }{1-\tau }A_{k}\left(p_{k}\left(N-1\right)+P_{\max}\right)}+\xi \left(P_{\max}-\sum_{i=1}^{K}p_{i}\right) \end{array}$$

where ξ is the Lagrange multiplier associated with constraint $\sum_{k=1}^{K}p_{k}=P_{\max}$. The dual optimization problem of Equation (9) is defined as $\min_{\xi \geq 0}\max_{p⪰0}\;L\left(p,\xi \right)$, then the optimal $p_{k}$ can be obtained by the first order optimal condition by the following equation.

(A4) $$\frac{\partial L}{\partial p_{k}}=\frac{A_{k}N\tau \left(1+\frac{\tau }{1-\tau }A_{k}P_{\max}\right)}{{\left(1+\frac{\tau }{1-\tau }A_{k}\left(p_{k}\left(N-1\right)+P_{\max}\right)\right)}^{2}}-\xi =0$$

Because $\frac{\partial^{2}L}{\partial p_{k}^{2}}=\frac{-2A_{k}^{2}N\left(N-1\right)\tau^{2}\left(1+\frac{\tau }{1-\tau }A_{k}P_{\max}\right)}{\left(1-\tau \right){\left(1+\frac{\tau }{1-\tau }A_{k}\left(p_{k}\left(N-\right)+P_{\max}\right)\right)}^{3}}<0$ and $p_{i}\geq 0,i=1,..K,$ From Equation (A4) and we have

(A5) $$p_{k}=\frac{1}{N-1}{\left(\left(\sqrt{\frac{A_{k}N\tau \left(1+\frac{\tau }{1-\tau }A_{k}P_{\max}\right)}{\xi }}-1\right)\frac{1-\tau }{A_{k}\tau }-P_{\max}\right)}^{+}$$

Substitute Equation (A5) into the $\sum_{k=1}^{K}p_{k}=P_{\max}$, then ξ can be obtained by the solution to the following equation:

(A6) $$\sum_{k=1}^{K}\frac{{\left(\left(\sqrt{\frac{A_{k}N\tau \left(1+\frac{\tau }{1-\tau }A_{k}P_{\max}\right)}{\xi }}-1\right)\frac{1-\tau }{A_{k}\tau }-P_{\max}\right)}^{+}}{N-1}=P_{\max}$$

Thus, Theorem 1 is proved. □

**Proof** **(Proof** **of** **Theorem** **2).** To prove Theorem 2, we first give two lemmas as follows.

**Lemma**  **A1.**
*$p_{k}>0$ is held if and only if $\xi <b_{k}\left(\tau \right)$ is satisfied.*

**Proof** **(Proof of Lemma A1).** Because $p_{k}=\frac{1}{N-1}{\left(\left(\sqrt{\frac{A_{k}N\tau \left(1+\frac{\tau }{1-\tau }A_{k}P_{\max}\right)}{\xi }}-1\right)\frac{1-\tau }{A_{k}\tau }-P_{\max}\right)}^{+}$ from Equation (10), we know $p_{k}>0$ is held if only if the following inequality is held.

(A7) $$\left(\sqrt{\frac{A_{k}N\tau \left(1+\frac{\tau }{1-\tau }A_{k}P_{\max}\right)}{\xi }}-1\right)\frac{1-\tau }{A_{k}\tau }>P_{\max}$$

(A7) is held if and only if the following inequality is held.

(A8) $$\sqrt{\frac{A_{k}N\tau \left(1+\frac{\tau }{1-\tau }A_{k}P_{\max}\right)}{\xi }}>\frac{P_{\max}A_{k}\tau }{1-\tau }+1$$

(A8) is held if and only if the following inequality is held.

(A9) $$\frac{A_{k}N\tau \left(1+\frac{\tau }{1-\tau }A_{k}P_{\max}\right)}{\xi }>{\left(\frac{P_{\max}A_{k}\tau }{1-\tau }+1\right)}^{2}$$

(A8) is held is if and only if ξ satisfies the following condition.

(A10) $$\xi <\frac{A_{k}N\tau \left(1+\frac{\tau }{1-\tau }A_{k}P_{\max}\right)}{{\left(\frac{P_{\max}A_{k}\tau }{1-\tau }+1\right)}^{2}}=\frac{A_{k}N\tau }{\left(\frac{P_{\max}A_{k}\tau }{1-\tau }+1\right)}=b_{k}\left(\tau \right)$$

Thus, Lemma A1 is held. □

**Lemma** **A2.**
*Let $p_{1},\cdots ,p_{K}$ be the optimal solution to Equation *(9)*, if $p_{i}>0$ is held, then $p_{j}>0$ is held for all j>i.*

**Proof** **(Proof of Lemma A2)** From Lemma A1, we have $\xi <b_{i}\left(\tau \right)$ is held if $p_{i}>0$ is held. Moreover, as the SNs are sorted such that $A_{1}\leq A_{2}.\cdots \leq A_{K}$ and $b_{j}\left(\tau \right)=\frac{A_{j}N\tau }{\left(\frac{P_{\max}A_{j}\tau }{1-\tau }+1\right)},j=1,\cdots ,K,$ then we have $b_{1}\left(\tau \right)\leq \cdots \leq b_{K}\left(\tau \right)$. Therefore, $\xi <b_{j}\left(\tau \right)$ is held for all j>i. Using Lemma A1, we have $p_{j}>0$ is held for all j>i. Thus, Lemma A2 is proved. □

Let $p_{1},\cdots ,p_{K}$ be the optimal solution to Equation (9) and $i=\min_{1\leq j\leq K}\left\{j|p_{j}>0\right\}$ be the first number of sensor node whose power is larger than zero, by Lemma A1 we know that $i=\min_{1\leq j\leq K}\left\{j|p_{j}>0\right\}$ if only if $b_{i-1}\left(\tau \right)\leq \xi <b_{i}\left(\tau \right)$ is held, where we define $b_{i-1}=-\infty$ when i=1. Moreover, using Lemma A2, we can simplify the equation in Equation (11) as follows.

(A11) $$\sum_{k=i}^{K}\frac{\left(\left(\sqrt{\frac{A_{k}N\tau \left(1+\frac{\tau }{1-\tau }A_{k}P_{\max}\right)}{\xi }}-1\right)\frac{1-\tau }{A_{k}\tau }-P_{\max}\right)}{N-1}=P_{\max}$$

(A11) can be rewritten as follows.

(A12) $$\frac{\sum_{k=i}^{K}\frac{\sqrt{A_{k}N\tau \left(1+\frac{\tau }{1-\tau }A_{k}P_{\max}\right)}}{\sqrt{\xi }}-\sum_{k=i}^{K}\frac{1-\tau }{A_{k}\tau }-\left(K-i+1\right){P_{\max}}^{}}{N-1}=P_{\max}$$

From (A12), we can obtain the closed-form solution ξ as follows.

(A13) $$\xi =\frac{{\left(\sum_{k=i}^{K}\sqrt{A_{k}N\tau \left(1+\frac{\tau }{1-\tau }A_{k}P_{\max}\right)}\frac{1-\tau }{A_{k}\tau }\right)}^{2}}{{\left(\left(K-i+N\right)P_{\max}+\sum_{k=i}^{K}\frac{1-\tau }{A_{k}\tau }\right)}^{2}}=a_{i}\left(\tau \right)$$

Therefore, when $b_{i-1}\left(\tau \right)\leq a_{i}\left(\tau \right)<b_{i}\left(\tau \right)$ is held, the closed-form solution ξ is given by (A13). Because $i=\min_{1\leq j\leq K}\left\{j|p_{j}>0\right\}$ if only if $b_{i-1}\left(\tau \right)\leq \xi <b_{i}\left(\tau \right)$ and $\xi =a_{i}\left(\tau \right)$ are held, we can rewritten *i* as $i=\min_{1\leq j\leq K}\left\{j|a_{j}\left(\tau \right)<b_{j}\left(\tau \right)\right\}$, $a_{j}\left(\tau \right)$. Thus, Theorem 2 is proved. □
